# A213 UNDERSTANDING PATIENT AND PHYSICIAN ATTITUDES AND EXPECTATIONS REGARDING IDENTIFYING AND MANAGING ANXIETY AND DEPRESSION IN IBD

**DOI:** 10.1093/jcag/gwac036.213

**Published:** 2023-03-07

**Authors:** A Patel, L Targownik, S Zelinsky, K Daley, L Jeffs, L Zeng, S Tabatabavakili

**Affiliations:** 1 Gastroenterology, Mount Sinai Hospital, Toronto; 2 Alberta SPOR Support Unit, Calgary; 3 IMAGINE SPOR Network, Hamilton; 4 Mount Sinai Hospital; 5 University of Toronto, Toronto, Canada

## Abstract

**Background:**

Patients living with Inflammatory Bowel Disease (IBD) commonly experience a number of mental health-related challenges, specifically anxiety and mood disorders (AMDs). Although there has been an awareness of the relationship between IBD and AMD within the GI research and clinical space; detection, treatment, and management amongst care providers is limited. Therefore, we are seeking to explore the overall experiences of patients living with Inflammatory Bowel Disease to identify and evaluate their experiences in interactions with GI clinicians around mental health in diverse care settings in order to determine how to best support mental health care amongst IBD patients.

**Purpose:**

We aimed to explore perspectives, experiences and barriers to engaging with mental health-related challenges amongst IBD patients when interacting with gastroenterologists over the course of their health journey.

**Method:**

We conducted 5 semi-structured online focus groups co-facilitated by patient researchers in early 2020 through *Zoom* which spanned for a total of 2.5 hours each. Participants were recruited through social media channels, GI clinics, the IMAGINE-SPOR unit, and Crohn’s and Colitis Canada. A semi-structured interview guide was developed for patient researchers to follow during the focus groups which provided guided questions that would allow patient participants to explore and reflect on: their experiences living with IBD, their expectations around mental health support, their perception of the engagement of GIs in mental health discussions, and their expectations for mental health support and services moving forward. Audio recordings from the semi-structured focus groups were then transcribed and thematic analysis was used to identify emerging themes and patient expectations.

**Result(s):**

We identified the following key themes: 1) experiences with IBD: difficulties related to reintegrating into social settings, feelings of loneliness; 2) expectations around mental health support: the need to develop their own resiliency strategies due to the lack of structural resources regarding mental health and IBD in the clinical space; 3) GI engagement: HCPs were dismissive of mental health symptoms, often gaslighting patients when mentioning mental health concerns during clinical encounters; and 4) expectations: a need to standardize mental health care across IBD care practice with a focus on potentially integrating healthcare providers of diverse care settings to help address the need for mental health support in such a vast patient population.

**Conclusion(s):**

Our study suggests that effective detection, management and awareness, along with the integration of feedback from patient lived experiences can help inform the development of mental health support and services which cater to the needs of people living with IBD. Results from this study will be interpreted in line with insight gathered from upcoming interviews of gastroenterologists and HCPs.

**Please acknowledge all funding agencies by checking the applicable boxes below:**

CIHR, Other

**Please indicate your source of funding;:**

IMAGINE SPOR INCUBATOR Grant

**Disclosure of Interest:**

None Declared

